# Internalized Stigma and Its Relationship With Quality of Life and Perceived Health Status in Rosacea and Acne Vulgaris: A Comparative Cross-Sectional Study

**DOI:** 10.7759/cureus.60731

**Published:** 2024-05-21

**Authors:** Esra Kıratlı Nalbant, Işıl Göğem İmren, Gamze Tas Dolek

**Affiliations:** 1 Dermatology, Ankara Yildirim Beyazıt University, Faculty of Medicine, Ankara, TUR; 2 Dermatology, Denizli State Hospital, Denizli, TUR; 3 Psychiatry, Sincan Dr. Nafiz Korez State Hospital, Ankara, TUR

**Keywords:** stigmatization level, stigma, rosacea, internalized stigma, acne

## Abstract

Background

Internalized stigma (IS) is the acceptance of unfavorable stereotypes about the disease that society has created and withdrawing from society with feelings of shame and worthlessness. Due to the visibility feature of facial skin diseases such as rosacea and acne vulgaris (AV), it is possible for them to cause IS.

Objective

We aimed to examine the level of IS in AV and rosacea patients and compare these patient groups as well.

Methods

Fifty-three AV and 46 rosacea patients aged 18-65 were included in this study. Internalized Stigma Scale (ISS) adapted for AV and rosacea were applied to all the patients. They also responded to the “Dermatology Life Quality Index” (DLQI) questionnaire. The relationship between IS levels and DLQI was investigated, and then acne and rosacea patients were compared with each other.

Results

Total DLQI, total ISS, and its subscales’ scores of all patients were found to be positively correlated with each other. When rosacea and AV patients were compared with each other, there was no difference regarding DLQI and ISS scores.

Conclusion

AV and rosacea patients experience high IS, and it is significantly related to low quality of life and health status. They also have similar IS levels when compared with each other.

## Introduction

Stigmatization is defined as a loss of social status and discrimination in particular societies, triggered by negative stereotypes associated with certain human characteristics. Stigma can harm people by affecting their internal perceptions, beliefs, and emotions. Internalized stigma (IS) manifests as the adoption of these negative stereotypes by the person themselves, resulting in feelings of worthlessness, shame, secrecy, and withdrawal [1,2].

Dermatological diseases that can be noticed from the outside have a feature that makes the individual different from others, which stigmatizes the patient. Patients may fear other people's reactions and experience serious self-confidence problems due to misperceptions about their illness [3,4]. Patients may even withdraw from society by accepting these misperceptions about themselves. All of these show that they actually experience IS. In social life, the first and most important body part we use to communicate with other people is our face. Therefore, any visible problem in this area becomes more important. Acne vulgaris (AV) and rosacea are the most common dermatological diseases affecting the face, and the psychological burden and IS of these diseases have only just begun to be investigated in the literature. Although IS has been investigated in patients with acne in the literature, there are very few studies on this subject in patients with rosacea [5-8]. Additionally, rosacea is a less well-known entity than acne among the public. Therefore, it is possible for patients to be labeled as alcoholics due to the erythema on their faces [9]. They may experience more IS than acne patients.

In this study, we aimed to examine the level of IS in AV and rosacea patients and to discover its relationship with quality of life and Perceived Health Status. We also aimed to compare these patient groups and identify the impact of demographic and clinical characteristics on IS levels.

## Materials and methods

The study was planned as a descriptive, multi-center, cross-sectional study. Fifty-three AV and 46 rosacea patients aged 18-65 were included in this study. Exclusion criteria for this study are having a communication disability, being pregnant or on a breastfeeding period, having psychiatric disease or any other systemic diseases, or having had any trauma, accident, or surgery that could cause a dysmorphic appearance. The ethical approval for this study was received from the local ethical committee (protocol number: E-60116787-020-178412; date of approval: 12/14/2021).

Sociodemographic features of the patients, including age, gender, smoking status, alcohol use, and clinical features of AV and rosacea, were recorded. Disease duration, rosacea type (papulopustular, erythematotelangiectatic, phymatous, or ocular), and disease severity were also recorded. The investigators described the assessment of disease severity separately by using a standardized Global Acne Severity Scale (GEA Scale) for AV patients and the Investigator Global Assessment (IGA) scale for rosacea patients [10,11]. “ISS” adapted for AV and rosacea, was applied to all the patients. Patients also responded to the “Perceived Health Status” and “Dermatology Life Quality Index (DLQI)” questionnaires.

Questionnaires and scales

Internalized Stigma Scale (ISS)

ISS, first developed for mental illnesses in 2003, [12] was utilized for the first time in dermatology by Alpsoy et al. in 2015 [13]. It is a Likert-type scale comprising 29 particular questions that measure the IS levels. The scale includes five subscales: alienation, stereotype endorsements, perceived discrimination, social withdrawal, and stigma resistance. Higher scores mean that the person's IS is more severe in the negative direction. The ISS score was calculated with the particular scale points for each item. However, items of the stigma resistance subscale were added reversely. The total scores may vary in the range of 29-116 [14].

Dermatology Life Quality Index (DLQI)

DLQI includes 10 questions concerning patients' apprehension of the impact of skin diseases on different angles of their health-related quality of life over the last week. Total scores can range from 0 to 30: high scores indicate poor quality of life [15].

Perceived Health Status Questionnaire

Perceived Health Status (PHS) is a Likert-type scale investigating general health by using a single question [16]. Scale scores were sorted out as “very good: 5,” “good: 4,” “medium: 3,” “bad: 2,” and “very bad: 1.”

Statistical methods

The normality of data distribution was assessed using the single-sample Kolmogorov-Smirnov test. For comparing the normal distribution or skew distribution variables between the two groups of independent samples, the student t-test or the Mann-Whitney U test were used. Categorical data were analyzed using the Pearson χ^2^ test or Fisher’s Exact test. Propensity score-matched analysis (PSM) was conducted to reduce the effects of bias using a logistic regression with a caliper value of 0.01, incorporating patients’ age and gender. A 1:1 Acne: Rosacea group matching was done using PSM. p<0.05 was considered as significant.

## Results

Fifty-three AV (female, n = 27; male, n = 26) and 46 rosacea (female, n = 35; male = 11) were included in the study. The mean ages of AV and rosacea patients were 24.42 ± 6.98 and 39.17 ± 10.36, respectively. The mean age of all patients was 31.27 ± 11.39. The mean disease durations were 6.85 ± 4.97 for AV, 7.78 ± 5.84 for rosacea, and 7.28 ± 5.39 for all patients. The rosacea group consisted of 16 (34.8%) patients with papulopustular type rosacea (PPR), 24 (52.2%) patients with end treatment response (ETR), five (10.9%) patients with phymatous, and one (2.1%) patient with ocular. When all rosacea patients were evaluated according to disease severity, 15 (32.6%) had mild rosacea, 26 (56.5%) had moderate, and five (10.9%) had severe or very severe disease. Of all AV patients, 20 (37.7%) had mild, 22 (41.5%) had moderate, and 11 (20.8%) had severe disease (Table 1).

**Table 1:** Demographic and Clinical Features of All Patients AV: acne vulgaris; PPR: papulopustular type rosacea; ETR: erythematotelangiectatic type rosacea; DLQI: Dermatology Life Quality Index; ISS: Internalized Stigma Scale Score; PHS: Perceived Health Status Point

Characteristic	AV (N=53)c	Rosacea (N=46)	All patients (N=99)
Age (mean ±SD)	24.42 ± 6.98	39.17 ± 10.36	31.27 ± 11.39
Gender, n (%)			
Male	26 (49.1%)	11 (23.9%)	37 (37.4%)
Female	27 (50.9%)	35 (76.1%)	62 (62.6%)
Disease Duration (mean ±SD)	6.85 ± 4.97	7.78 ± 5.84	7.28 ± 5.39
Rosacea Type, n (%)			
PPR		16 (34.8%)	
ETR		24 (52.2%)	
Ocular		1 (2.2%)	
Phymatosis		5 (10.9%)	
Disease Severity, n (%)			
1	20 (37.7%)	15 (32.6%)	35 (35.4%)
2	22 (41.5%)	26 (56.5%)	48 (48.5%)
3	11 (20.8%)	5 (10.9%)	16 (16.2%)
DLQI (mean ± SD)	8.81 ± 6.39	8.07 ± 6.1	8.46 ± 6.24
ISS total (mean ± SD)	54.87 ± 12.07	56.33 ± 12.99	55.55 ± 12.46
PHS, n (%)			
1,2,3	29 (54.7%)	28 (60.9%)	57 (57.5%)
4,5	24 (45.3%)	18 (39.1%)	42 (42.5%)

The mean total ISS score and DLQI were found to be 55.55 ± 12.46, and 8.46 ± 6.24 for all patients. Of all patients, PHS of %57.5 was in the range of 1-3 (worse than good), %42.5 was 4-5 (good). Total DLQI and ISS scores had no significant correlation with age, gender, or disease duration. But disease severity was positively correlated with ISS total (r = 0.225, p = 0.025) and DLQI score (r = 0.206, p = 0.041). DLQI, ISS total score and its subscales were found to be positively correlated with each other (r values were between 0.319 and 0.937; all p values were < 0.05) (Table 2).

**Table 2:** Correlation of Data, All Patients DLQI: Dermatology Life Quality Index; ISS: Internalized Stigma Scale Score; PHS: Perceived Health Status Statistically analyzed with Spearman Correlation test.

Characteristic	DLQI		ISS Total		PHS	
	Rho	p-value	Rho	p-value	Rho	p-value
Age	0.028	0.786	0.119	0.243	0.034	0.739
Gender	0.037	0.719	0.048	0.635	-0.104	0.306
Disease duration	0.13	0.201	0.079	0.438	0.054	0.597
Disease severity	0.206	0.041	0.225	0.025	- 0.031	0.758
ISS Subscales						
Alienation	0.645	0.001	0.88	0.001	- 0.391	0.001
Stereotype endorsement	0.48	0.001	0.863	0.001	-0.2	0.048
Perceived discrimination	0.418	0.001	0.854	0.001	-0.155	0.125
Social withdrawal	0.606	0.001	0.937	0.001	-0.357	0.001
Stigma resistance	0.319	0.001	0.409	0.006	-0.332	0.001
DLQI			0.658	0.001	-0.66	0.001
ISS total	0.658	0.001			-0.373	0.001
PHS	-0.66	0.001	-0.373	0.001		

The mean total DLQI and ISS scores of AV patients were 8.81 ± 6.39 and 54.87 ± 12.07, respectively (Table 1). When the AV group was evaluated within itself, total DLQI and ISS scores had no significant correlation with age, gender, or disease duration. On the other hand, disease severity was positively correlated with the ISS total score (r = 0.295, p = 0.032). Additionally, statistically significantly positive correlations were discovered between DLQI and ISS total (r = 0.661, P <.001) and ISS subscales (r values were between 0.352 and 0.894; all p values were < 0.05). Besides, there were negative correlations between PHS and DLQI (r = -0.738, p<0.001) and ISS total (r = -0.441, p<0.001) (Table 3).

**Table 3:** Correlation of Data, AV Patients DLQI: Dermatology Life Quality Index; ISS: Internalized Stigma Scale Score; PHS: Perceived Health Status; AV: acne vulgaris Statistically analyzed with Spearman Correlation test.

Characteristic	DLQI		ISS Total	
	rho	p-value	Rho	p-value
Age	0.122	0.384	0.09	0.5
Gender	-0.059	0.673	-0.04	0.75
Disease duration	0.069	0.623	0.022	0.87
Disease severity	0.233	0.094	0.295	0.032
The ISS Subscales				
Alienation	0.623	0.001	0.894	0.001
Stereotype endorsement	0.515	0.001	0.842	0.001
Perceived discrimination	0.317	0.021	0.801	0.001
Social withdrawal	0.584	0.001	0.911	0.001
Stigma resistance	0.352	0.01	0.414	0.002
DLQI			0.661	0.001
ISS total	0.661	0.001		
PHS	-0.738	0.001	-0.441	0.001

The median total DLQI and ISS scores of rosacea patients were 8.07 ± 6.1 and 56.33 ± 12.99, respectively (Table 1). When the rosacea group was examined within itself, total ISS and DLQI had no significant correlation with age, gender, disease duration, or disease severity. Besides, statistically significant positive correlations were detected between DLQI and ISS total (r = 0.665, P <.001) and ISS subscales (r values were between 0.453 and 0.667; all p values were < 0.05) except stigma resistance subscale (r = 0.29, p = 0.051). Besides, PHS was negatively correlated with DLQI (r = -0.563, p<0.001) and ISS total (r = -0.298, p<0.001) (Table 4).

**Table 4:** Correlation of Data, Rosacea Patients DLQI: Dermatology Life Quality Index; ISS: Internalized Stigma Scale Score; PHS: Perceived Health Status Statistically analyzed with Spearman Correlation test.

Characteristic	DLQI		ISS Total	
	Rho	p-value	rho	p-value
Age	-0.026	0.862	0.034	0.824
Gender	0.21	0.161	0.131	0.387
Disease duration	0.18	0.23	0.119	0.43
Disease severity	0.153	0.309	0.153	0.312
The ISS Subscales				
Alienation	0.667	0.001	0.839	0.001
Stereotype endorsement	0.453	0.002	0.888	0.001
Perceived discrimination	0.53	0.001	0.906	0.001
Social withdrawal	0.638	0.001	0.948	0.001
Stigma resistance	0.29	0.051	0.396	0.006
DLQI			0.665	0.001
ISS	0.665	0.001		
PHS	-0.563	0.001	-0.298	0.044

After PSM analysis was conducted to match patients’ age and gender; rosacea and AV patients were compared with each other. As a result, no substantial difference was found between the two disease groups in terms of DLQI, ISS total, and PHS scores (Table 5).

**Table 5:** Comparison of AV and Rosacea Patient Groups after Propensity Score Matching AV: acne vulgaris; DLQI: Dermatology Life Quality Index; ISS: Internalized Stigma Scale Score; PHS: Perceived Health Status *Statistically analyzed with Student t-test ^#^Fisher's Exact test ^$^Pearson Chi-Square test; others analyzed with Mann-Whitney U test.

Characteristic	AV (N:28) (median, IQR)	Rosacea (N:28) (median, IQR)	p-value
Age*	30 (8.75)	33.5 (4.75)	0.298
Gender^#^			0.121
Male	10 (35.7)	4 (14.3)	
Female	18(64.3)	24 (85.7)	
DLQI	10 (11)	8 (9)	0.237
ISS Total*	56.5 (16)	56 (24)	0.828
The ISS Subscales			
Alienation	13 (5)	13 (6)	0.934
Stereotype endorsement	12 (4)	11 (4)	0.267
Perceived discrimination	8.5 (2)	8 (3)	0.351
Social withdrawal	11 (5)	10.5 (7)	0.418
Stigma resistance	12.5 (5)	13 (4)	0.447
PHS^$^			0.428
1	3 (10.7)	0 (0)	
2	5 (17.9)	5 (17.9)	
3	9 (32.1)	13 (46.4)	
4	6 (21.4)	6 (21.4)	
5	5 (17.9)	4 (14.3)	

## Discussion

In this study, we discovered high levels of IS in both AV and rosacea patients, showing a parallelism between poor quality of life and PHS. These findings support previous studies in this area [5-8]. It was found that IS was not affected by age, gender, or disease duration in these patients. On the other hand, it has been shown that IS increases with disease severity.

We detected no significant difference in the IS levels of AV patients in terms of age, gender, or disease duration. In the literature, the relationship between gender and IS seems to be inconsistent. Ogut also found no relationship between these features and IS levels, similar to our findings [17]. On the contrary, while Adkins et al. reported higher stigmatization levels in female AV patients [7], Kotekoglu et al. detected higher levels of IS in male AV patients. Additionally, they demonstrated that IS levels were not affected by age [6]. In this study, we found that IS levels increase with acne severity, consistent with previous studies [5,17]. We also found no difference in IS levels regarding age and gender in rosacea patients. In a study examining feelings of stigmatization in rosacea patients, the authors found that the frequency of perceived stigmatization is higher in younger adults and males [8]. This may be because they used a different survey to determine perceived stigmatization than the scale we used. On the other hand, they found a positive correlation between the duration of rosacea and stigmatization, which supports our findings. In our study, we also observed that disease severity and IS levels are not associated with each other in rosacea, unlike in AV. This finding shows that patients with rosacea experience high levels of IS, even if their disease is not severe. This could be because the rosacea itself has a more chronic course than AV.

A recent large study on stigmatization in patients with various skin diseases, including acne and rosacea, revealed that these patients reported feeling rejected by others and receiving disgusting looks [18]. Besides, having a visible skin disease significantly reduces patients' quality of life [19]. In this study, we demonstrated a strong correlation between the levels of IS that patients experience and their poor quality of life. It has been revealed that these patients feel different from society due to their illness, withdraw themselves from social life, and feel ashamed and worthless. As a result, their quality of life and their general health perception were negatively affected. The psychological burden of acne is well studied in the literature. The current study reinforces the findings from the study by Davern and O'Donnell, which emphasizes that perceived stigma predicts health-related quality of life and psychological distress in acne patients [5]. In our study, we have also focused on rosacea patients and compared these patient groups. It was shown that the patients with rosacea were affected at least as much as patients with acne, regardless of age and gender.

Limitations

We must comment on two issues that could potentially limit the scope of our study. First, we did not question the patients about their socioeconomic status. Socioeconomic status can also have an impact on the patients' psychology and IS levels. Second, this study lacks a control group because the ISS scale's disease-related questions do not apply to healthy individuals. Therefore, there is a need to conduct further studies using different scales that are also applicable to healthy individuals.

## Conclusions

Patients with AV and rosacea experience high IS, and the clinician must never forget the psychological burden of these diseases. When compared within themselves, both AV and rosacea experience similar IS levels. However, in contrast to AV, IS levels in rosacea are high regardless of disease severity. As a result, the clinician should also prioritize the psychological status of these patients, as well as their clinical severity.
